# Heat waves in the Colombian Caribbean: A public health problem?

**DOI:** 10.15649/cuidarte.3199

**Published:** 2024-05-23

**Authors:** Oskarly Pérez-Anaya, Jorge Homero Wilches-Visbal, Lídice Álvarez-Miño

**Affiliations:** 1 Facultad de Ciencias de la Salud, Universidad del Magdalena, Santa Marta, Colombia. operez@unimagdalena.edu.co Universidad del Magdalena Facultad de Ciencias de la Salud Universidad del Magdalena Santa Marta Colombia operez@unimagdalena.edu.co; 2 Facultad de Ciencias de la Salud, Universidad del Magdalena, Santa Marta, Colombia. jwilches@unimagdalena.edu.co Universidad del Magdalena Facultad de Ciencias de la Salud Universidad del Magdalena Santa Marta Colombia jwilches@unimagdalena.edu.co; 3 Facultad de Ciencias de la Salud, Universidad del Magdalena, Santa Marta, Colombia. lalvarezm@unimagdalena.edu.co Universidad del Magdalena Facultad de Ciencias de la Salud Universidad del Magdalena Santa Marta Colombia lalvarezm@unimagdalena.edu.co

A popular belief states that if frog is submerged in a container and gradually heats it up, it will try to adapt until it dies; this is probably the situation faced by more and more human populations. As stated by thousands of scientists, academics, and researchers worldwide, the planet’s warming is directly related to climate change.

Throughout history, human activities have significantly impacted the environment, ecosystems, and plant and animal diversity, which existed long before the first Homo sapiens appeared^1^. One of the phenomena attributed to this activity is anthropogenic climate change. According to the United Nations^2^, climate change is a variation of climate caused directly or indirectly by human activity that tends to alter the composition of the Earth’s atmosphere. In this sense, it is a global phenomenon, and its main signal is the increase in temperature resulting from the trapping of the sun’s rays in the dense layer of greenhouse gases. This layer consists mainly of carbon dioxide, methane, and nitrous oxide and ultimately causes the warming of all layers of the atmosphere in general, including the troposphere^3^. It is currently estimated that the planet’s average temperature will increase by about 3.2 °C (37.76 °F) this century, severely affecting human health^4^.

The World Health Organization has warned that about 150,000 people worldwide die each year due to climate change; it predicts that between 2030 and 2050, climate change will cause more than 250,000 deaths each year due to malnutrition, diarrhea, malaria, and heat waves^1^^,^^5^. To better understand the relationship between climate change and human health, the effects have been divided into three groups: (1) Water and food scarcity, leading to disruption of agricultural production systems, conflicts, etc.; (2) direct impacts on health and well-being, such as displacement, mortality from various preventable causes, mental health problems, increased infectious diseases, etc.; and (3) impacts on cities such as floods, storms, damage to key economic infrastructure, etc^6^.

One of the events attributed to climate change is heat waves, which have increased in frequency and intensity in recent decades. Although there is no consensus on the definition of “heat wave,” it is certain that some of the concepts vary from region to region and country to country since it is a phenomenon that is temporarily manifested in a specific region. For example, in some areas of the United States, a heat wave occurs when a temperature of at least 26.^7^ °C (80 °F) is recorded for at least 48 consecutive hours. In Australia, a heat wave occurs when the maximum temperature exceeds 35 °C (95 °F) for three or more consecutive days. In South Africa, a heat wave occurs when maximum temperatures exceed the 90th percentile for at least three consecutive days^7^.

The Colombian Institute of Hydrology, Meteorology and Environmental Studies (IDEAM for its acronym in Spanish) has defined a heat wave as a sequence or series of consecutive days during which the highest temperatures have remained above certain critical limits, defined as the values corresponding to the upper tercile of the historical series^8^. It is therefore recognized that the definition of a heat wave is determined regionally, establishing the normal daily temperature range according to the historical average, to identify those moments when this critical range is exceeded for a certain period, which can be at least two consecutive days.

With this in mind, it is worth asking whether we are living in constant heat waves locally. According to historical reports from government agencies in the region^9^, heat waves usually occur between April and August, with a peak in May. But does the scientific evidence support this perception? To answer this question, three variables are analyzed: average air temperature, thermal comfort level and average wind speed in the city of Santa Marta. The data for the variables were retrieved from IDEAM and the specialized weather web portal WeatherSpark (https://es.weatherspark.com/).

**Figure 1:**
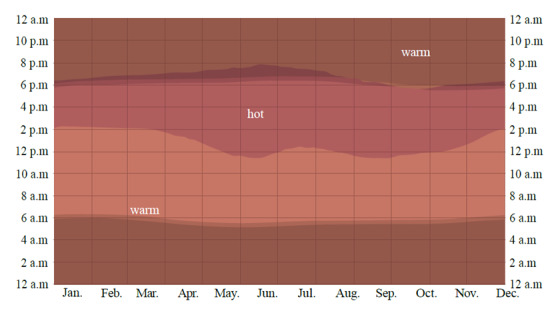
Historical average hourly temperature during the year in Santa Marta (2015-2023)

According to the most recent thermal data for Santa Marta (2015-2023), the annual average temperature is 27.9 °C (82.22 °F) (SD = 0.67), with an annual average low temperature of 25.5 °C (77.9 °F) (SD = 1.1) and an annual average high temperature of 31.0 °C (87.8 °F) (SD = 0.0). May and June have the highest low (27 °C [80.6 °F]) and average (29 °C [84.2 °F]) temperatures of the year^10^. The months with the longest daily period of very hot temperatures are May-June with 7 to 8 hours above 29 °C (84.2 °F) between 12 pm and 7 pm (Figure 1).

Warm: 75-84 °F (Tmean = 78.8 °F); hot: 85-95 °F (Tmean= 89.6 °F). Source: ©https://es.weatherspark.com/ ^10^.

**Figure 2:**
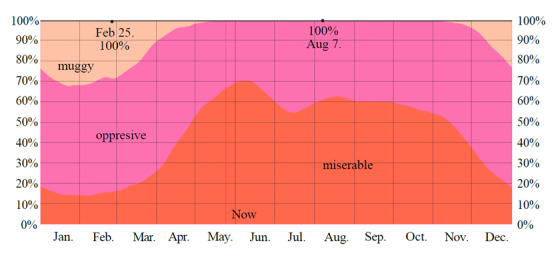
Level of humidity comfort per day during the year in Santa Marta (2015 - 2023)

In addition, the thermal comfort level reported by WeatherSpark10 in May and June is “miserable” in 50-70% of the hours of the day (Figure 2).

Classified by dew point (DP) as: miserable: DP > 75 °F; oppressive: 70 < DP < 75 °F and muggy: 65 < DP < 70 °F. Source: ©https://es.weatherspark.com/ ^10^.

Thus, it could be said that on about 60% of the days in May and June, the heat index (HI) varies from 34 °C (93.2 °F) (T = 29 °C [84.2 °F] and DP = 24 °C [75.2 °F]) to 42 °C (107.6 °F) (T = 35 °C [95 °F] and DP = 24 °C [75.2 °F])^11^. The corresponding classifications are extreme caution (32 - 38 °C [89.6 - 100.4 °F]) and danger (39 - 51 °C [102.2 - 123.8 °F]). In these cases, cramps or heat exhaustion may occur due to prolonged exposure or physical activity^12^.

In 2022, IDEAM reported 26 days -12 in May and 14 in June- with maximum temperatures between 34 °C and 37 °C (93.2 °F and 98.6 °F), higher than the multiannual average high temperature (33 °C [91.4 °F]). Most of these temperatures occurred in the first and third weeks of each month^13^, exceeding the heat wave thresholds discussed earlier. The highest recorded temperature (37°C [98.6 °F]) occurred in the first week of May^13^. Assuming that IDEAM’s historical relative humidity data for that period14 did not change significantly by 2022, the 26-day HI is above 46 °C [114.8 °F]^11^. In any case, this HI is above the threshold for heatstroke or death from hyperthermia^15^. In the face of such climatic event, authors like Chesini et al.^16^ recommend that the competent authorities (municipal, departmental, and national) focus their attention on older adults, children, chronically ill individuals, and those who work in environments that are exposed to heat waves. In addition, several studies suggest that heat waves may increase hospitalizations, mortality, and the relative risk of cardiovascular, cerebrovascular, respiratory, and renal disease. It should be noted that there is physiological evidence of a relationship between elevated temperatures and increased blood pressure, blood viscosity, and heart rate.

In addition, Salazar-Ceballos and Álvarez-Miño^17^ propose a joint development with the environment, giving priority to green spaces where physical activity can be practiced; therefore, infrastructure planning and health promotion should be the foundation of cities, with the aim of reducing urban heat islands. The authors of this document also recommend issuing a yellow alert in the main affected municipalities and adopting recommendations related to: Hydration areas, change in working hours (between 10 a.m. and 3 p.m.), restriction of physical activity in the same period of the day, maintenance of ventilation in public transport vehicles, emergency brigades for electric utilities, partial financing of utility bills, and a waiting period of at least five minutes before entering closed areas where air conditioning is on, to avoid possible respiratory illnesses that could lead to a collapse of the health system or, in the worst case, to death.
